# Assessment of the complexity of renal tumors by nephrometry (R.E.N.A.L. score) with CT and MRI images versus 3D reconstruction model images

**DOI:** 10.1590/S1677-5538.IBJU.2020.0930

**Published:** 2021-07-20

**Authors:** Tadeu J. F. L. Campos, Francisco E. de V., Marcos F. H. Rocha

**Affiliations:** 1 Hospital Geral de Fortaleza FortalezaCE Brasil Hospital Geral de Fortaleza, Fortaleza, CE, Brasil.

## INTRODUCTION

In recent years, with greater population access to imaging tests, the profile of renal neoplasia has changed, with increased diagnosis being made in the early stages (1).

In this new scenario, it became necessary to popularize alternative treatment modalities to radical nephrectomy to avoid overtreatment and its possible consequences. Radical nephrectomy is associated with a higher global mortality, mainly from cardiovascular causes, due to the long-term increased loss of renal function (2). In this context, partial nephrectomy gained strength, as studies showed cancer safety similar to radical nephrectomy, associated with a greater overall survival (3).

With technical advances, partial nephrectomy, which had initially been applied only to small kidney lesions, commenced in increasingly larger and more complex kidney tumors (4).

Thus, there was a growing interest in partial nephrectomy based on studies proving the oncological efficacy of this technique and the gain in overall survival with nephron-sparing surgery (5).

In this new era, nephrometry systems were developed to predict the feasibility of partial nephrectomy in the face of complex renal lesions. Among these, the most frequently used are the R.E.N.A.L. (radius; exophytic/endophytic; nearness; anterior/posterior; location) and P.A.D.U.A. (preoperative aspects and dimensions used for anatomic) scores (6).

The most widely used nephrometric system is the R.E.N.A.L. score, and despite being defined based on objective parameters, a frequent discrepancy in scores between different examiners, radiologists, and urologists exists, especially when considering different quality image examinations (7).

The R.E.N.A.L. score, conceived in principle to be evaluated using simple tomographic images, is now also evaluated in three-dimensional (3D) reconstruction models.

There is a perception that the R.E.N.A.L. score assessed in 3D reconstructions is more favorable to the performance of partial nephrectomy than the assessments made from simple tomography or resonance images (8).

The present study proposed to assess whether there were different interpretations of the R.E.N.A.L. score by radiologists and urologists, when evaluated from simple computed tomography (CT) or magnetic resonance imaging (MRI) findings, and from 3D reconstructions. Finally, we also aimed to assess whether this possible different interpretation may imply decision-making regarding the performance of partial or radical nephrectomy.

**Figure 1:**
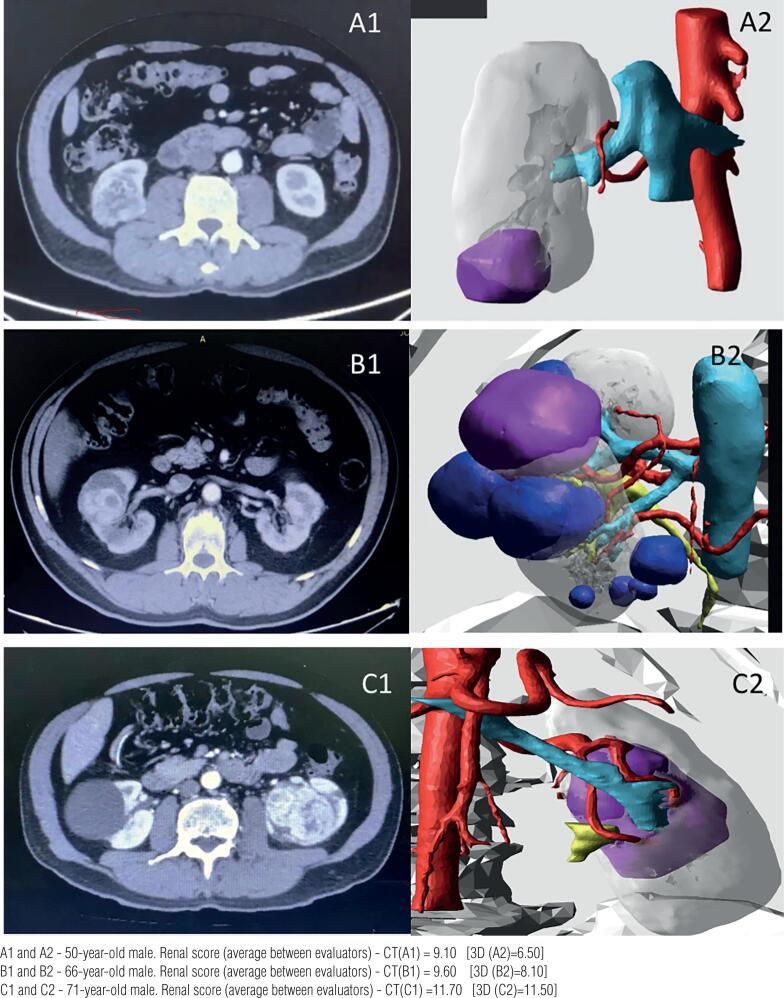
Illustration and comparison some cases of patients with renal masses undergoing 3D reconstruction before partial nephrectomy.

Figure-1 illustrates and compares some cases of patients with renal masses undergoing 3D reconstruction before partial nephrectomy.

## MATERIALS AND METHODS

The study was carried out based on the analysis of simple CT or MRI images, and after 3D reconstruction of patients with renal nodules who underwent partial or radical nephrectomy.

The study was conducted on eight tomography/resonance examinations and the respective 3D reconstructions of different patients undergoing nephrectomy. Each examination was evaluated separately by five radiologists and five urologists, with a total of 160 evaluations.

The 3D reconstructions were obtained using the DocDo application of the Brazilian company InfiniBrains^®^.

Initially, the radiologist/urologist was asked to rate the R.E.N.A.L. score from simple CT or MRI images. Following this, the score was assessed based on the 3D reconstructed images of the renal nodule of the same patient.

The radiologist/urologist evaluated the simple CT or MRI images without identifying the patients and in a different and random order from the 3D reconstructions.

At the end of the evaluations, statistical analyses of the incidence of different interpretations of the R.E.N.A.L. score among the radiologists and urologists using different technologies were conducted.

For this purpose, the statistical analysis program “GraphPad Prism” was used, using the “D’Agostino and Pearson” normality test, followed by the t-test or the Mann-Whitney test to analyze the correlations between the variables.

Finally, the potential effects of the different interpretations of the R.E.N.A.L. score on surgical decision-making in patients with complex renal nodules was discussed.

The study was conducted in accordance with national and international laws and was approved by the institutional ethics committee (protocol no. 4.264.545).

## RESULTS

The study was conducted on eight patients who underwent partial or radical nephrectomy of renal nodules. Each patient was evaluated by five radiologists and five urologists participating in the study.

3D reconstructions of the images obtained during the examinations of all patients were performed. Six patients underwent simple CT, and two patients underwent MRI. All examinations were performed using contrast.

The evaluator, radiologist, or urologist, scored the R.E.N.A.L. score for each patient, primarily from the simple CT or MRI images, and then from 3D reconstructions.

For data analysis, the statistical analysis program “GraphPad Prism” was used, using the D’ Agostino-Pearson normality test, followed by t-test or Mann-Whitney test to analyze the correlations between variables. The results of this analysis.

After analyzing the results, it was clearly observed that in most evaluations, there was a different interpretation of the R.E.N.A.L. score, by the same evaluator, when simple CT or MRI images, and 3D reconstructions were compared. In 81% (65 out of 80) evaluations, the R.E.N.A.L. score differed between simple CT or MRI images, and 3D reconstructions of the same patient (Figure-1).

Out of the 65 evaluations in which the score was different between 3D reconstruction and simple images of the same patient, five (8%) evaluations differed only in the anterior or posterior parameter, with no difference in the numerical result of the score. In 16 (25%) evaluations, simple CT or MRI images had a lower score compared to reconstruction. Finally, in 44 assessments, which corresponded to 67%, the score was lower after assessment of the 3D reconstruction.

After statistical analysis of each patient separately, including the 10 evaluations by radiologists and urologists, a statistical difference in the R.E.N.A.L. score was observed in three patients when comparing simple images and reconstructions.

Patients with statistical differences in scores were considered to have tumors of intermediate complexity, with an average score between 7 and 9, corresponding to 37.5% of the total.

For patients with tumors of lesser and greater complexity, there were no statistically significant differences between the simple images and the reconstructions, although in all patients, the average 3D reconstruction score was lower than the average score of the simple CT and MRI scans (Table-1).

## DISCUSSION

Partial nephrectomy has gained a prominent role in the treatment of kidney cancer due to the diagnosis of increasingly smaller tumors and studies proving the oncological efficacy of this procedure combined with better overall survival (9).

Nephron-sparing surgery is associated with less long-term loss of kidney function compared to radical nephrectomies. As a result, partial nephrectomy determines a lower cardiovascular risk, and is therefore the standard treatment when feasible (5).

Three-dimensional reconstructions have emerged as a technological tool with great potential to facilitate the performance of partial nephrectomy. Reconstruction helps in surgical planning, predicting and anticipating difficulties, decreasing the rate of complications, and increasing the success rate (10).

Several studies have evaluated the role of 3D reconstruction as an auxiliary tool for partial nephrectomy. The performance of partial nephrectomy in complex tumors is challenging because of the need for satisfactory preservation of the parenchyma, reduced ischemia time, and oncological safety of surgical margins.

Some studies have shown that 3D reconstruction allows more precise surgical planning with selective vascular clamping, resulting in smaller areas of ischemia, and greater preservation of nephrons, contributing to better preservation of renal function.

In a study by Wang et al., it was concluded that for complex tumors with a R.E.N.A.L. score greater than 8, the use of 3D reconstruction was associated with a shorter ischemia time and greater preservation of renal parenchyma, resulting in better preservation of renal function (11).

According to Ukimura et al., in complex renal masses, 3D images accurately identified arterial branches and facilitated partial nephrectomy with zero ischemia (12).

Mercader et al. reported an experience using a patient-specific 3D-printed renal tumor model for surgical planning of a complex heminephrectomy in a horseshoe kidney and found that it was useful for easier surgical planning (13).

Minervini et al. analyzed the use of intraoperative ultrasonography and 3D-virtual models and found that it improved the perception of tumor anatomy and vascularization, maximizing outcomes (14).

In addition, 3D reconstruction has been used as an adjuvant in percutaneous nephrolithotripsy. Bianchi et al. observed that it may be helpful to reduce operative time and improve the learning curve (15).

The R.E.N.A.L. score, used to assess complexity of renal nodules and to predict complications of partial nephrectomy, is often used as an auxiliary tool to indicate or contraindicate nephron-sparing surgery (16).

In this sense, 3D reconstruction with the potential to assess the R.E.N.A.L. score appears to be an important tool to increase the rate of partial nephrectomy.

The present study showed that in 37.5% of the patients, there was a statistically significant difference in the R.E.N.A.L. score between the simple images and the reconstructions, which could interfere in the conduct of these patients regarding the performance of partial nephrectomy.

It was observed that the patients who had a statistical difference were those who had tumors of intermediate complexity, suggesting that for these tumors, 3D reconstruction has a more important role in the possibility of interfering in the conduct.

For extreme tumors, that is, of high and low complexity, it seems that 3D reconstruction should not interfere with the performance of partial nephrectomy. However, as mentioned above, some studies have already shown the important role of 3D reconstruction in the planning and success of partial nephrectomies in more complex tumors.

Finally, even for patients in whom the 3D reconstruction showed no statistical difference in the R.E.N.A.L. score, it is still of fundamental importance in surgical planning (even if it does not interfere with the conduct regarding the performance of partial or radical nephrectomy), as it improves the anatomical understanding of the relationship between the tumor in the kidney, and the vascular structures and excretory pathway.

Thus, we can suggest that 3D reconstruction is of great importance in renal nodules for which the possibility of partial nephrectomy is being considered, as it tends to be used progressively.

## CONCLUSIONS

Partial nephrectomy has a prominent role in the current treatment of renal cancer. The importance of technologies facilitating this procedure is clear. The 3D reconstructions of imaging examinations are of great value for better planning of partial nephrectomy.

In this study, we observed that the three-dimensional reconstruction changed the perception of the R.E.N.A.L. score by the evaluators, with a statistical difference in tumors of intermediate complexity in this sample.

Three-dimensional reconstruction has emerged as a new tool that tends to be increasingly used with the potential to interfere in conduct, increasing the rate of partial nephrectomy or, at least, facilitate surgical planning, decrease the rate of complications, and increase the rate of success.

## ABBREVIATIONS

3D: Three dimensional
CT: Computed tomography
MRI: Magnetic resonance imaging
